# Synthesis and biological evaluation of histamine Schiff bases as carbonic anhydrase I, II, IV, VII, and IX activators

**DOI:** 10.1080/14756366.2017.1386660

**Published:** 2017-10-26

**Authors:** Suleyman Akocak, Nabih Lolak, Daniela Vullo, Mustafa Durgun, Claudiu T. Supuran

**Affiliations:** a Department of Pharmaceutical Chemistry, Faculty of Pharmacy, Adiyaman University, Adiyaman, Turkey;; b NEUROFARBA Dept., Sezione di Scienze Farmaceutiche, Università degli Studi di Firenze, Florence, Italy;; c Department of Chemistry, Faculty of Science and Literature, Harran University, Sanliurfa, Turkey

**Keywords:** Carbonic anhydrase activators, histamine, Schiff bases, isozymes, Alzheimer’s disease

## Abstract

A series of 20 histamine Schiff base was synthesised by reaction of histamine, a well known carbonic anhydrase (CA, E.C 4.2.2.1.) activator pharmacophore, with substituted aldehydes. The obtained histamine Schiff bases were assayed as activators of five selected human (h) CA isozymes, the cytosolic hCA I, hCA II, and hCA VII, the membrane-anchored hCA IV and transmembrane hCA IX. Some of these compounds showed efficient activity (in the nanomolar range) against the cytosolic isoform hCA VII, which is a key CA enzyme involved in brain metabolism. Moderate activity was observed against hCA I and hCA IV (in the nanomolar to low micromolar range). The structure–activity relationship for activation of these isoforms with the new histamine Schiff bases is discussed in detail based on the nature of the aliphatic, aromatic, or heterocyclic moiety present in the aldehyde fragment of the molecule, which may participate in diverse interactions with amino acid residues at the entrance of the active site, where activators bind, and which is the most variable part among the different CA isoforms.

## Introduction

Carbonic anhydrases (CAs, EC 4.2.1.1) are zinc containing metalloenzymes (present in prokaryotes and eukaryotes) that catalyse the reversible hydration of carbon dioxide into bicarbonate and proton ions under physiological conditions (CO_2_+H_2_O ↔ HCO_3_^–^+H^+^). Up to now, seven genetically distinct CA families (α-, β-, γ-, δ-, ζ-, η-, and θ-CAs), as well as numerous isoforms in most organisms were discovered^1–7^. In humans, 16 different CA and CA related proteins have been described, with different subcellular localisation, catalytic activity, and susceptibility to different classes of inhibitors and activators^8–12^. Some of these isoforms are cytosolic (CA I, CA II, CA III, CA VII, and CA XIII), some of them are transmembrane bound isoforms (CA IV, CA IX, CA XII, CA XIV, and CA XV), two of them are mitochondrial (CA VA and CA VB), and one of them is secreted in saliva and milk (CA VI). On the other hand, catalytically inactive CA related isoforms (CARP VIII, CARP X, and CA XI) are also cytosolic proteins^1–4^^,^^8^^,^^9^^,^^11^^,^^13–15^.

Carbonic anhydrase has been a therapeutic target for many years and their inhibitors are clinically used/investigated as diuretics, anticonvulsant, antiobesity, antiglaucoma and more recently antitumour and anti-infective agents^16–19^. However, the CA activators (CAAs), although investigated simultaneously with inhibitors, do not have pharmaceutical applications, yet. Indeed, it has been proposed that some CAAs might have applications in the neurodegenerative disorder of memory and cognitive function (Alzheimer’s disease) since it has been shown the level of brain CAs significantly diminished in the brain of Alzheimer’s disease and older rats as compared to normal and young brain of animals^20^^,^^21^.

**Figure 1.**
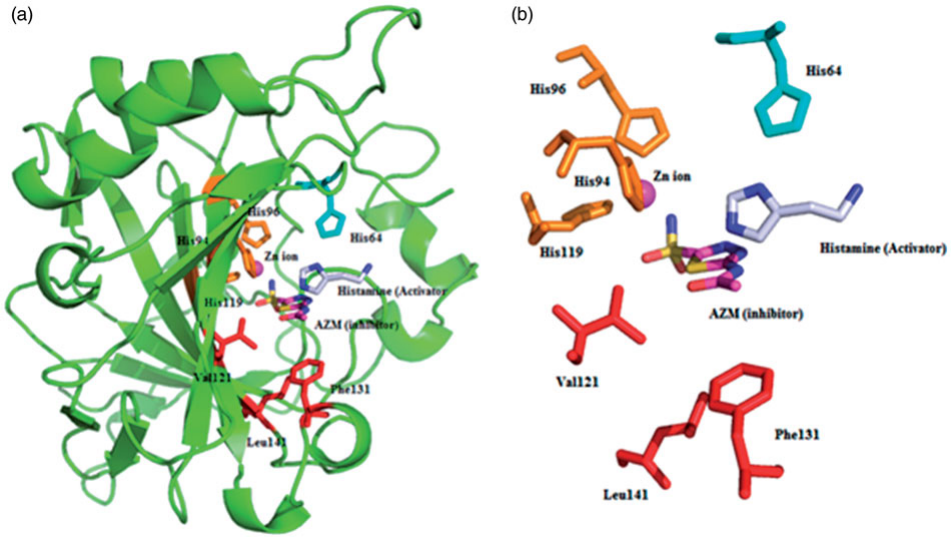
Superimposed ribbon diagram (a) and active site detail (b) of the CA II isozyme (PDB codes 1AVN^22^ and 3HS4^37^) with the activator histamine and the well known CAI 5-acetamido-1,3,4-thiadiazole-2-sulphonamide (acetazolamide, AZM). Acetazolamide is coordinated to the zinc ion being bound deep within the active site, whereas histamine does not interact with the metal ion and is bound at the entrance of the cavity. The zinc ion (magenta) is coordinated by three histidine residues (His 94, His 96, and His 119, in orange) and some key amino acids on active site were shown (in red). The proton shuttle residue His 64 is also shown (in cyan). Figure made using PyMol (DeLano Scientific).

The inhibition and activation processes of CAs are well investigated processes which show different binding modes within the active site cavity of isozymes (Figure 1)^11^^,^^22^. The classical inhibitors bind deep within the active site cavity by interacting with the metal centre, which is zinc ion for hCAs (Figure 1(b)). On the other hand, activators are bound far away from the metal ion, at the entrance-middle part of the active site cavity and participate in the proton shuttling process from the active site to the external buffer. This is part of the normal catalytic cycle of the enzyme, with the amino acid residue His 64 in isoforms such as CA II, IV, VI, VII, IX, XII, found in the middle of the active site cavity, normally participating in this step, which is the rate determining one for the entire catalytic cycle (Figure 1(a))^22^^,^^23^. The presence of the activators produces an alternative proton transfer pathway and enhances the overall catalytic efficiency of the enzyme. CAAs play thus a role on speeding up the deprotonation of zinc bound water, which decrease the pKa of a coordinated water molecule, in the rate determining step of the catalytic mechanism (Equation (2) with the generation of the active form of the enzyme^24–36^, as described in Equation (3) where the formation of the enzyme–activator complex is shown, which leads to an increased rate of the proton transfer reaction due to the fact that the process became intermolecular and not intramolecular as in Equation (2).

(1) $${\text{EZn}}^{\text{2+}}{\text{-OH}}^{-}{\text{+CO}}_{\text{2}}\;⇌\;{\text{EZn}}^{\text{2+}}{\text{-HCO}}_{{\text{3}}^{\text{-}}}\overset{{\text{+H}}_{\text{2}}\text{O}}{⇌}{\text{EZn}}^{\text{2+}}{\text{-OH}}_{\text{2}}{\text{+HCO}}_{{\text{3}}^{-}}$$

(2) $${\text{ENn}}^{\text{2}}{\text{-OH}}_{\text{2}}\;⇌\;{\text{EZn}}^{\text{2+}}{\text{-OH}}^{-}{\text{+H}}^{\text{+}}\text{-rate}\;\text{determining}\;\text{step-}$$

(3) $$\begin{array}{l} {\text{ENn}}^{\text{2}}{\text{-OH}}_{\text{2}}\;+\text{A}\;⇌\;\;[{\text{EZn}}^{\text{2+}}{\text{-OH}}_{{\text{2}}^{\text{-}}}\text{A]}\;⇌\;[{\text{EZn}}^{\text{2+}}{\text{-OH}}^{-}{\text{-AH}}^{\text{+}}\text{]}\\ ⇌\;[{\text{EZn}}^{\text{2+}}{\text{-OH}}^{-}{\text{-AH}}^{+}\text{]}\;⇌\;{\text{EZn}}^{\text{2+}}{\text{-OH}}^{-}{\text{-AH}}^{+} \end{array}$$

In order to possess a good activity, a compound needs both steric requirements (i.e. to fit within the restricted active site cavity) and electronic factors (to possess an appropriate pKa value of the proton shuttle moiety) to be present in its structure. The X-ray crystal structure for the hCA II-histamine adduct^22^ revealed that the imidazole moiety is bound in the middle of the cavity, not far from the natural proton shuttle residue (His 64), whereas the amino moiety is not involved in any interaction with the enzyme active site and that is why this amino group might be derivatised to obtain much more biologically active compounds, as done earlier by some of us.

Schiff base derivatives of sulphonamides were extensively studied as an efficient and selective inhibitors of several CAs by us and other researchers^38–41^. However, a procedure successfully used to obtain sulphonamide CAIs incorporating Schiff base moieties, that is, reaction of amino sulphonamide with aldehydes to produce Schiff base derivatives, has never been approached for preparing histamine derivatives with potential CA activating properties.

Herein, we present the synthesis and CA activation studies of histamine Schiff base derivatives on a panel of selected CA isozymes which are cytosolic hCA I, II, VII (involved in many physiological processes all over the body and also in the brain)^1–3^ and membrane-bound ones hCA IV and IX (the last of which is a validated anti-tumour target)^16–18^.

## Materials and methods

### General

All chemicals and anhydrous solvents were purchased from Sigma-Aldrich, Merck, Alfa Aesar and TCI and used without further purification. Melting points (mp) were determined with SMP30 melting point apparatus in open capillaries and are uncorrected. Elemental analysis was carried out on a LEO CHNS model 932 elemental analyser. FT-IR spectra were recorded by using Perkin Elmer Spectrum 100 FT-IR spectrometer. Nuclear magnetic resonance (^1^H NMR and ^13^C NMR) spectra of compounds were recorded using a Bruker Advance III 300 MHz spectrometer in DMSO-d_6_ and TMS as an internal standard operating at 300 MHz for ^1^H NMR and 75 MHz for ^13^C NMR. Thin layer chromatography (TLC) was carried out on Merck silica gel 60 F_254_ plates.

### General procedure for the synthesis of histamine Schiff bases H (1–20)

The potassium hydroxide (10 mmol) was added to a stirred suspension of histamine dihydrochloride (5 mmol) in dry MeOH (10–15 ml) at room temperature. After stirring for 2 h, the precipitate salt (KCl) was filtered off and the filtrate was treated with a solution of aldehydes (5 mmol) in dry MeOH (20–25 ml). The homogeneous mixture was stirred overnight at room temperature. The completion of the reaction was monitored by TLC and FT-IR. The excess solvent was evaporated and the oily residue was crystallised with ethyl acetate and ether to obtain corresponding Schiff base derivatives. The desired final products **H(1–20)** were dried under vacuum and fully characterised by FT-IR, ^1^H NMR, ^13^C NMR, elemental analysis, and melting points.

**2-(1H-imidazol-4-yl)-N-pentylideneethanamine (H1):** Yield: 55%; colour: white powder, mp: 188–190 °C: FT-IR (cm^−1^): 1648 (–C=N–); ^1^H NMR (DMSO-d_6_, 300 MHz, *δ* ppm): 8.45 (s, 1H, –N=CH–), 7.75 (d, 1H, *J* = 1.2, H-2 Im), 7.30 (s, 1H, H-5 Im), 3.62 (t, 2H, *J* = 6.5, –CH_2_CH_2_-Im), 2.88 (t, 2H, *J* = 6.5, –CH_2_CH_2_-Im), 1.65 (t, 2H, Val), 1.30 (m, 2H, Val), 1.24 (m, 2H, Val), 0.9 (t, 3H, Val): ^13^C NMR (DMSO-d_6_, 75 MHz, *δ* ppm): 164.38 (–N=CH–), 142.35, 138.63, 118.31, 63.53, 35.13, 29.16, 28.32, 23.25, 14.58; elemental analysis for C_10_H_17_N_3_: C, 67.00; H, 9.56; N, 23.44. Found: C, 66.98; H, 9.55; N, 23.46.

**N-(furan-2-ylmethylene)-2-(1H-imidazol-4-yl)ethanamine (H2):** Yield: 45%; colour: brown powder, mp: 202–205 °C; FT-IR (cm^−1^): 1645 (–C=N–); ^1^H NMR (DMSO-d_6_, 300 MHz, *δ* ppm): 8.78 (s, 1H, –N=CH–), 7.93 (d, 1H, *J* = 1.2, H-2 Im), 7.82 (d, 1H, *J* = 2.2, furan), 7.74 (s, 1H, H-5 Im), 6.98 (d, 1H, *J* = 2.2, furan), 6.72 (d, 1H, *J* = 2.0, furan) 3.48 (t, 2H, *J* = 6.0, –CH_2_CH_2_-Im), 2.87 (t, 2H, *J* = 6.0, –CH_2_CH_2_-Im): ^13^C NMR (DMSO-d_6_, 75 MHz, *δ* ppm): 165.71 (–N=CH–), 151.15, 145.63, 138.42, 134.18, 119.62, 113.34, 56.15, 32.10; elemental analysis for C_10_H_11_N_3_O: C, 63.48; H, 5.86; N, 22.21. Found: C, 63.45; H, 5.85; N, 22.24.

**2-(1H-imidazol-4-yl)-N-(4-methylbenzylidene)ethanamine (H3):** Yield: 72%; colour: white powder, mp: 190–193 °C; FT-IR (cm^−1^): 1640 (–C=N–); ^1^H NMR (DMSO-d_6_, 300 MHz, *δ* ppm): 8.62 (s, 1H, –N=CH–), 7.88 (d, 1H, *J* = 1.0, H-2 Im), 7.78 (d, 2H, *J* = 8.2, Ar-H), 7.35 (s, 1H, H-5 Im), 7.28 (d, 2H, *J* = 8.2, Ar-H), 3.65 (t, 2H, *J* = 6.2, –CH_2_CH_2_-Im), 2.90 (t, 2H, *J* = 6.2, –CH_2_CH_2_-Im), 1.85 (s, 3H, –CH_3_): ^13^C NMR (DMSO-d_6_, 75 MHz, *δ* ppm): 166.41 (–N=CH–), 163.82, 133.51, 132.93, 131.65, 116.31, 115.64, 114.73, 111.42, 55.24, 26.72, 18.38; elemental analysis for C_13_H_15_N_3_: C, 73.21; H, 7.09; N, 19.70. Found: C, 73.19; H, 7.10; N, 19.73.

**2-(1H-imidazol-4-yl)-N-(4-methoxybenzylidene)ethanamine (H4):** Yield: 78%; colour: light brown powder, mp: 178–181 °C; FT-IR (cm^−1^): 1636 (–C=N–); ^1^H NMR (DMSO-d_6_, 300 MHz, *δ* ppm): 8.66 (s, 1H, –N=CH–), 7.92 (d, 1H, *J* = 1.2, H-2 Im), 7.82 (d, 2H, *J* = 7.8, Ar-H), 7.42 (s, 1H, H-5 Im), 7.33 (d, 2H, *J* = 7.8, Ar-H), 3.85 (s, 3H, –OCH_3_), 3.62 (t, 2H, *J* = 6.5, –CH_2_CH_2_-Im), 2.92 (t, 2H, *J* = 6.5, –CH_2_CH_2_-Im): ^13^C NMR (DMSO-d_6_, 75 MHz, *δ* ppm): 167.33 (–N=CH–), 162.62, 134.48, 133.35, 131.83, 116.68, 115.38, 112.32, 56.65, 54.98, 26.35; elemental analysis for C_13_H_15_N_3_O: C, 68.10; H, 6.59; N, 18.33. Found: C, 68.08; H, 6.60; N, 18.35.

**4-(((2-(1H-imidazol-4-yl)ethyl)imino)methyl)-N,N-dimethylaniline (H5):** Yield: 73%; colour: light red powder, mp: 194–196 °C; FT-IR (cm^−1^): 1638 (–C=N–); ^1^H NMR (DMSO-d_6_, 300 MHz, *δ* ppm): 8.76 (s, 1H, –N=CH–), 7.95 (d, 1H, *J* = 0.9, H-2 Im), 7.86 (d, 2H, *J* = 8.2, Ar-H), 7.45 (s, 1H, H-5 Im), 7.28 (d, 2H, *J* = 8.2, Ar-H), 3.70 (t, 2H, *J* = 6.6, –CH_2_CH_2_-Im), 3.35 (s, 6H, –N(CH_3_)_2_): 2.95 (t, 2H, *J* = 6.6, –CH_2_CH_2_-Im): ^13^C NMR (DMSO-d_6_, 75 MHz, *δ* ppm): 166.35 (–N=CH–), 162.48, 134.83, 133.35, 131.83, 116.68, 115.38, 112.32, 56.65, 54.98, 26.35; elemental analysis for C_14_H_18_N_4_: C, 69.39; H, 7.49; N, 23.12. Found: C, 69.40; H, 7.45; N, 23.15.

**2-(((2-(1H-imidazol-4-yl)ethyl)imino)methyl)phenol (H6):** Yield: 88%; colour: light yellow powder, mp: 180–182 °C; FT-IR (cm^−1^): 1640 (–C=N–); ^1^H NMR (DMSO-d_6_, 300 MHz, *δ* ppm): 14.42 (s, 1H, –OH), 8.49 (s, 1H, –N=CH–), 7.78 (d, 1H, *J* = 1.0, H-2 Im), 7.60–7.57 (d, 1H, *J* = 8.2, Ar-H), 7.31–7.28 (d, 1H, *J* = 783, Ar-H), 6.88 (s, 1H, H-5 Im), 6.81–6.74 (m, 2H, Ar-H), 3.78 (t, 2H, *J* = 6.8, –CH_2_CH_2_-Im), 2.88 (t, 2H, *J* = 6.8, –CH_2_CH_2_-Im): ^13^C NMR (DMSO-d_6_, 75 MHz, *δ* ppm): 163.18 (–N=CH–), 160.42, 132.11, 131.22, 130.12, 115.48, 115.10, 114.78, 111.15, 54.83, 26.25; elemental analysis for C_12_H_13_N_3_O: C, 66.96; H, 6.09; N, 19.52. Found: C, 66.98; H, 6.05; N, 19.55.

**2-(((2-(1H-imidazol-4-yl)ethyl)imino)methyl)-6-methylphenol (H7):** Yield: 78%; colour: white powder, mp: 168–171 °C; FT-IR (cm^−1^): 1636 (–C=N–); ^1^H NMR (DMSO-d_6_, 300 MHz, *δ* ppm): 14.45 (s, 1H, –OH), 8.53 (s, 1H, –N=CH–), 7.81 (d, 1H, *J* = 0.9, H-2 Im), 7.64–7.60 (d, 1H, *J* = 7.5, Ar-H), 7.38–7.35 (d, 1H, *J* = 7.5, Ar-H), 6.93 (s, 1H, H-5 Im), 6.60 (t, 1H, *J* = 10.8, Ar-H), 3.89 (t, 2H, *J* = 6.7, –CH_2_CH_2_-Im), 2.92 (t, 2H, *J* = 6.7, –CH_2_CH_2_-Im), 2.15 (s, 3H, –CH_3_): ^13^C NMR (DMSO-d_6_, 75 MHz, *δ* ppm): 165.38 (–N=CH–), 162.35, 133.57, 132.21, 131.78, 117.10, 116.23, 115.32, 112.13, 55.32, 25.15, 18.34; elemental analysis for C_13_H_15_N_3_O: C, 68.10; H, 6.59; N, 18.33. Found: C, 68.08; H, 6.60; N, 18.36.

**2-(((2-(1H-imidazol-4-yl)ethyl)imino)methyl)-6-methoxyphenol (H8):** Yield: 82%; colour: dark yellow powder, mp: 190–192 °C FT-IR (cm^−1^): 1632 (–C=N–); ^1^H NMR (DMSO-d_6_, 300 MHz, *δ* ppm): 14.46 (s, 1H, –OH), 8.51 (s, 1H, –N=CH–), 7.82 (d, 1H, *J* = 0.9, H-2 Im), 7.63–7.59 (d, 1H, *J* = 7.8, Ar-H), 7.35–7.32 (d, 1H, *J* = 7.8, Ar-H), 6.92 (s, 1H, H-5 Im), 6.58 (t, 1H, *J* = 10.8, Ar-H), 3.98 (s, 3H, –OCH_3_), 3.87 (t, 2H, *J* = 6.6, –CH_2_CH_2_-Im), 2.90 (t, 2H, *J* = 6.6, –CH_2_CH_2_-Im): ^13^C NMR (DMSO-d_6_, 75 MHz, *δ* ppm): 164.25 (–N=CH–), 161.65, 132.38, 131.24, 130.14, 116.82, 116.12, 115.44, 111.56, 56.32, 54.26, 27.54; elemental analysis for C_13_H_15_N_3_O_2_: C, 63.66; H, 6.16; N, 17.13. Found: C, 63.61; H, 6.18; N, 17.16.

**2-(((2-(1H-imidazol-4-yl)ethyl)imino)methyl)-6-bromophenol (H9):** Yield: 85%; colour: yellow powder, mp: 145–147 °C; FT-IR (cm^−1^): 1635 (–C=N–); ^1^H NMR (DMSO-d_6_, 300 MHz, *δ* ppm): 14.60 (s, 1H, –OH), 8.53 (s, 1H, –N=CH–), 7.85 (d, 1H, *J* = 0.9, H-2 Im), 7.64–7.60 (d, 1H, *J* = 7.8, Ar-H), 7.36–7.33 (d, 1H, *J* = 7.8, Ar-H), 6.96 (s, 1H, H-5 Im), 6.61 (t, 1H, *J* = 10.8, Ar-H), 3.92 (t, 2H, *J* = 6.6, –CH_2_CH_2_-Im), 2.92 (t, 2H, *J* = 6.6, –CH_2_CH_2_-Im): ^13^C NMR (DMSO-d_6_, 75 MHz, *δ* ppm): 166.13 (–N=CH–), 162.72, 136.46, 134.63, 133.61, 132.14, 117.58, 116.62, 116.41, 112.76, 54.28, 27.54; elemental analysis for C_12_H_12_BrN_3_O: C, 49.00; H, 4.11; N, 14.29. Found: C, 49.03; H, 4.08; N, 14.31.

**2-(((2-(1H-imidazol-4-yl)ethyl)imino)methyl)-4-bromophenol (H10):** Yield: 67%; colour: yellow powder, mp: 168–170 °C; FT-IR (cm^−1^): 1640 (–C=N–); ^1^H NMR (DMSO-d_6_, 300 MHz, *δ* ppm): 14.45 (s, 1H, –OH), 8.78 (s, 1H, –N=CH–), 7.88 (d, 1H, *J* = 1.0, H-2 Im), 7.72–7.68 (d, 1H, *J* = 8.2, Ar-H), 7.55–7.46 (m, 2H, Ar-H), 7.12 (s, 1H, H-5 Im), 3.95 (t, 2H, *J* = 6.8, –CH_2_CH_2_-Im), 2.95 (t, 2H, *J* = 6.8, –CH_2_CH_2_-Im): ^13^C NMR (DMSO-d_6_, 75 MHz, *δ* ppm): 167.48 (–N=CH–), 162.89, 136.45, 134.12, 133.54, 132.68, 117.45, 116.14, 115.58, 112.23, 55.57, 27.43; elemental analysis for C_12_H_12_BrN_3_O: C, 49.00; H, 4.11; N, 14.29. Found: C, 49.01; H, 4.06; N, 14.32.

**2-(((2-(1H-imidazol-4-yl)ethyl)imino)methyl)-4-chlorophenol (H11):** Yield: 70%; colour: light yellow powder, mp: 184–186 °C; FT-IR (cm^−1^): 1639 (–C=N–); ^1^H NMR (DMSO-d_6_, 300 MHz, *δ* ppm): 14.48 (s, 1H, –OH), 8.75 (s, 1H, –N=CH–), 7.84 (d, 1H, *J* = 1.0, H-2 Im), 7.70–7.67 (d, 1H, *J* = 8.2, Ar-H), 7.52–7.47 (m, 2H, Ar-H), 7.10 (s, 1H, H-5 Im), 3.92 (t, 2H, *J* = 6.8, –CH_2_CH_2_-Im), 2.91 (t, 2H, *J* = 6.8, –CH_2_CH_2_-Im): ^13^C NMR (DMSO-d_6_, 75 MHz, *δ* ppm): 166.95 (–N=CH–), 162.47, 136.32, 134.08, 133.38, 132.92, 117.12, 116.59, 115.46, 112.81, 56.12, 27.18; elemental analysis for C_12_H_12_ClN_3_O: C, 57.72; H, 4.84; N, 14.20. Found: C, 57.70; H, 4.80; N, 14.25.

**2-(((2-(1H-imidazol-4-yl)ethyl)imino)methyl)-4,6-dibromophenol (H12):** Yield: 62%; colour: yellow powder, mp: 165–167 °C; FT-IR (cm^−1^): 1643 (–C=N–); ^1^H NMR (DMSO-d_6_, 300 MHz, *δ* ppm): 14.68 (s, 1H, –OH), 8.82 (s, 1H, –N=CH–), 7.92 (d, 1H, *J* = 1.0, H-2 Im), 7.80 (s, 1H, Ar-H), 7.65 (s, 1H, Ar-H), 7.32 (s, 1H, H-5 Im), 3.98 (t, 2H, *J* = 6.6, –CH_2_CH_2_-Im), 2.96 (t, 2H, *J* = 6.6, –CH_2_CH_2_-Im): ^13^C NMR (DMSO-d_6_, 75 MHz, *δ* ppm): 168.12 (–N=CH–), 163.37, 137.28, 135.06, 134.42, 132.98, 117.65, 116.34, 115.80, 112.39, 56.63, 27.87; elemental analysis for C_12_H_11_Br_2_N_3_O: C, 38.64; H, 2.97; N, 11.26. Found: C, 38.60; H, 3.00; N, 11.31.

**2-(((2-(1H-imidazol-4-yl)ethyl)imino)methyl)-4,6-dichlorophenol (H13):** Yield: 60%; colour: yellow powder, mp: 134–136 °C; FT-IR (cm^−1^): 1642 (–C=N–); ^1^H NMR (DMSO-d_6_, 300 MHz, *δ* ppm): 14.70 (s, 1H, –OH), 8.80 (s, 1H, –N=CH–), 7.91 (d, 1H, *J* = 1.0, H-2 Im), 7.82 (s, 1H, Ar-H), 7.63 (s, 1H, Ar-H), 7.30 (s, 1H, H-5 Im), 3.96 (t, 2H, *J* = 6.6, –CH_2_CH_2_-Im), 2.94 (t, 2H, *J* = 6.6, –CH_2_CH_2_-Im): ^13^C NMR (DMSO-d_6_, 75 MHz, *δ* ppm): 168.05 (–N=CH–), 163.13, 137.69, 135.47, 134.43, 132.32, 117.38, 116.04, 115.86, 112.21, 56.38, 27.59; elemental analysis for C_12_H_11_Cl_2_N_3_O: C, 50.72; H, 3.90; N, 14.79. Found: C, 50.70; H, 3.92; N, 14.81.

**2-(1H-imidazol-4-yl)-N-((perfluorophenyl)methylene)ethanamine (H14):** Yield: 75%; colour: white powder, mp: 170–172 °C; FT-IR (cm^−1^): 1644 (–C=N–); ^1^H NMR (DMSO-d_6_, 300 MHz, *δ* ppm): 8.75 (s, 1H, –N=CH–), 7.92 (d, 1H, *J* = 1.0, H-2 Im), 7.32 (s, 1H, H-5 Im), 3.95 (t, 2H, *J* = 6.8, –CH_2_CH_2_-Im), 2.92 (t, 2H, *J* = 6.8, –CH_2_CH_2_-Im): ^13^C NMR (DMSO-d_6_, 75 MHz, *δ* ppm): 167.95 (–N=CH–), 163.37, 134.49, 133.12, 131.18, 130.67, 116.82, 115.89, 114.29, 111.95, 56.69, 27.92; elemental analysis for C_12_H_8_F_5_N_3_: C, 49.84; H, 2.79; N, 14.53. Found: C, 49.80; H, 2.80; N, 14.56.

**N-(2-bromobenzylidene)-2-(1H-imidazol-4-yl)ethanamine (H15):** Yield: 68%; colour: white powder, mp: 199–201 °C; FT-IR (cm^−1^): 1639 (–C=N–); ^1^H NMR (DMSO-d_6_, 300 MHz, *δ* ppm): 8.52 (s, 1H, –N=CH–), 7.75 (d, 1H, *J* = 1.2, H-2 Im), 7.63–7.59 (d, 1H, *J* = 7.8, Ar-H), 7.35–7.30 (d, 1H, *J* = 7.8, Ar-H), 6.92 (s, 1H, H-5 Im), 6.85–6.78 (m, 2H, Ar-H), 3.82 (t, 2H, *J* = 6.5, –CH_2_CH_2_-Im), 2.90 (t, 2H, *J* = 6.5, –CH_2_CH_2_-Im): ^13^C NMR (DMSO-d_6_, 75 MHz, *δ* ppm): 165.68 (–N=CH–), 161.45, 133.12, 131.88, 130.32, 130.58, 115.21, 115.01, 114.34, 111.55, 55.43, 27.42; elemental analysis for C_12_H_12_BrN_3_: C, 51.82; H, 4.35; N, 15.11. Found: C, 51.85; H, 4.30; N, 15.15.

**N-(5-bromo-2-methoxybenzylidene)-2-(1H-imidazol-4-yl)ethanamine (H16):** Yield: 72%; colour: white powder, mp: 204–206 °C; FT-IR (cm^−1^): 1641 (–C=N–); ^1^H NMR (DMSO-d_6_, 300 MHz, *δ* ppm): 8.75 (s, 1H, –N=CH–), 7.83 (d, 1H, *J* = 1.0, H-2 Im), 7.70–7.66 (d, 1H, *J* = 7.8, Ar-H), 7.52–7.45 (m, 2H, Ar-H), 7.21 (s, 1H, H-5 Im), 3.93 (t, 2H, *J* = 6.6, –CH_2_CH_2_-Im), 3.88 (s, 3H, –OCH_3_), 2.91 (t, 2H, *J* = 6.6, –CH_2_CH_2_-Im): ^13^C NMR (DMSO-d_6_, 75 MHz, *δ* ppm): 166.41 (–N=CH–), 161.79, 136.02, 134.45, 133.28, 132.78, 117.11, 116.41, 115.29, 112.72, 56.38, 55.93, 27.12; elemental analysis for C_13_H_14_BrN_3_O: C, 50.67; H, 4.58; N, 13.64. Found: C, 50.70; H, 4.55; N, 13.68.

**2-(((2-(1H-imidazol-4-yl)ethyl)imino)methyl)benzoic acid) (H17):** Yield: 68%; colour: cream powder, mp: 168–170 °C; FT-IR (cm^−1^): 1638 (–C=N–); ^1^H NMR (DMSO-d_6_, 300 MHz, *δ* ppm): 13.40 (br.s, 1H, –OH), 8.82 (s, 1H, –N=CH–), 8.12 (d, 1H, *J* = 0.9, H-2 Im), 7.90 (d, 1H, *J* = 6.5, Ar-H), 7.83–7.80 (m, 2H, Ar-H), 7.72–7.69 (d, 1H, *J* = 7.2, Ar-H), 7.12 (s, 1H, H-5 Im), 3.88 (t, 2H, *J* = 6.4, –CH_2_CH_2_-Im), 2.94 (t, 2H, *J* = 6.4, –CH_2_CH_2_-Im): ^13^C NMR (DMSO-d_6_, 75 MHz, *δ* ppm): 169.34 (–C=O), 163.72 (–N=CH–), 146.21, 139.38, 136.92, 133.72, 132.11, 130.48, 129.01, 127.43, 117.22, 59.32, 30.28; elemental analysis for C_13_H_13_N_3_O_2_: C, 64.19; H, 5.39; N, 17.27. Found: C, 64.15; H, 5.40; N, 17.30.

**2-(1H-imidazol-4-yl)-N-(4-isopropylbenzylidene)ethanamine (H18):** Yield: 74%; colour: white powder, mp: 196–198 °C; FT-IR (cm^−1^): 1634 (–C=N–); ^1^H NMR (DMSO-d_6_, 300 MHz, *δ* ppm): 8.72 (s, 1H, –N=CH–), 7.98 (d, 1H, *J* = 1.2, H-2 Im), 7.87 (d, 2H, *J* = 7.4, Ar-H), 7.48 (s, 1H, H-5 Im), 7.39 (d, 2H, *J* = 7.8, Ar-H), 3.78 (t, 2H, *J* = 6.2, –CH_2_CH_2_-Im), 2.95 (t, 2H, *J* = 6.2, –CH_2_CH_2_-Im), 2.82 (m, 1H, –CH(CH_3_)_2_), 1.28 (d, 6H, –CH(CH_3_)_2_): ^13^C NMR (DMSO-d_6_, 75 MHz, *δ* ppm): 165.31 (–N=CH–), 161.23, 135.78, 133.93, 132.36, 117.12, 115.65, 112.45, 57.84, 31.22, 28.65, 24.32; elemental analysis for C_15_H_19_N_3_: C, 74.65; H, 7.94; N, 17.41. Found: C, 74.61; H, 7.92; N, 17.45.

**4-(((2-(1H-imidazol-4-yl)ethyl)imino)methyl)benzonitrile (H19):** Yield: 85%; colour: white powder, mp: 188–191 °C; FT-IR (cm^−1^): 1638 (–C=N–); ^1^H NMR (DMSO-d_6_, 300 MHz, *δ* ppm): 8.78 (s, 1H, –N=CH–), 8.08 (d, 1H, *J* = 1.8, H-2 Im), 7.94 (d, 2H, *J* = 7.0, Ar-H), 7.54 (s, 1H, H-5 Im), 7.46 (d, 2H, *J* = 7.4, Ar-H), 3.88 (t, 2H, *J* = 6.4, –CH_2_CH_2_-Im), 2.98 (t, 2H, *J* = 6.4, –CH_2_CH_2_-Im): ^13^C NMR (DMSO-d_6_, 75 MHz, *δ* ppm): 165.31 (–N=CH–), 162.78, 139.38, 135.47, 133.28, 118.35, 116.56, 115.77, 112.42, 58.24, 27.85; elemental analysis for C_13_H_12_N_4_: C, 69.62; H, 5.39; N, 24.98. Found: C, 69.60; H, 5.35; N, 25.01.

**N-(4-bromo-2-methylbenzylidene)-2-(1H-imidazol-4-yl)ethanamine (H20):** Yield: 78%; colour: light yellow powder, mp: 208–210 °C; FT-IR (cm^−1^): 1640 (–C=N–); ^1^H NMR (DMSO-d_6_, 300 MHz, *δ* ppm): 8.88 (s, 1H, –N=CH–), 7.86 (d, 1H, *J* = 1.0, H-2 Im), 7.74–7.70 (d, 1H, *J* = 7.8, Ar-H), 7.58–7.52 (m, 2H, Ar-H), 7.33 (s, 1H, H-5 Im), 3.90 (t, 2H, *J* = 6.0, –CH_2_CH_2_-Im), 2.89 (t, 2H, *J* = 6.0, –CH_2_CH_2_-Im), 2.43 (s, 3H, –CH_3_): ^13^C NMR (DMSO-d_6_, 75 MHz, *δ* ppm): 165.67 (–N=CH–), 160.43, 136.85, 135.01, 133.69, 132.46, 117.58, 116.31, 115.79, 112.32, 57.11, 28.01, 19.44; elemental analysis for C_13_H_14_BrN_3_: C, 53.44; H, 4.83; N, 14.38. Found: C, 53.40; H, 4.85; N, 14.41.

### CA enzyme activation assay

An Sx.18Mv-R Applied Photophysics (Oxford, UK) stopped-flow instrument has been used to assay the catalytic activity of various CA isozymes for CO_2_ hydration reaction^42^. Phenol red (at a concentration of 0.2 mM) was used as indicator, working at the absorbance maximum of 557 nm, with 10 mM HEPES (pH 7.5) as buffer, 0.1 M Na_2_SO_4_ (for maintaining constant ionic strength), following the CA-catalysed CO_2_ hydration reaction for a period of 10 s at 25 °C. The CO_2_ concentrations ranged from 1.7 to 17 mM for the determination of the kinetic parameters and activation constants. For each activator at least six traces of the initial 5–10% of the reaction have been used for determining the initial velocity. The uncatalysed rates were determined in the same manner and subtracted from the total observed rates. Stock solutions of activators (10 mM) were prepared in distilled-deionised water and dilutions up to 0.001 mM were done thereafter with distilled-deionised water. Activator and enzyme solutions were pre-incubated together for 15 min (standard assay at room temperature, or for prolonged periods of 24–72 h, at 4 °C) prior to assay, in order to allow for the formation of the E–A complex. The activation constant (*K*_A_), defined similarly with the inhibition constant *K*_I_, can be obtained by considering the classical Michaelis–Menten equation (Equation (4), which has been fitted by non-linear least squares by using PRISM 3):

(4) $$v=v_{\max}/\{1+K_{\text{M}}/\left[S\right](1+{\left[A\right]}_{\text{f}}/K_{\text{A}})\}$$

where [*A*]_f_ is the free concentration of activator.

Working at substrate concentrations considerably lower than *K*_M_ ([*S*]** ≪ ***K*_M_), and considering that [*A*]_f_ can be represented in the form of the total concentration of the enzyme ([*E*]_t_) and activator ([*A*]_t_), the obtained competitive steady-state equation for determining the activation constant is given by Equation (5):

(5) $$v=v_{0}.K_{\text{A}}/\{K_{\text{A}}+({\left[A\right]}_{\text{t}}-0.\text{5}{\left\{({\left[A\right]}_{\text{t}}+{\left[E\right]}_{\text{t}}+K_{\text{A}})-{\left({\left[A\right]}_{\text{t}}+{\left[E\right]}_{\text{t}}+K_{\text{A}}\right)}^{\text{2}}-\text{4}{\left[A\right]}_{\text{t}}.{\left[E\right]}_{\text{t}}\right)}^{\text{1}/\text{2}}\}\}$$

where *v*_0_ represents the initial velocity of the enzyme-catalysed reaction in the absence of activator^19^^,^^25^^,^^40^^,^^43–46^.

## Results and discussion

### Chemistry

The rationale for designing new CAAs presented in this work is based on previous data which showed efficient CA activating effects for derivatised histamine based compounds. Furthermore, as mentioned above, the X-ray crystal structure for the hCA II-histamine adduct has been used to infer the fact that derivatisation of the amino moiety of histamine with not interfere with the binding of the compound in the CAA binding site^22–24^^,^^28^. According to X-ray results, the amino ethyl moiety of histamine does not have any interactions with enzyme and it might be derivatised in such a way as to make new contacts within the active site of the enzyme, which may lead to more efficient activators, as already demonstrated for sulphonylated, carboxamide and ureido derivatives of histamine^22–25^^,^^27^^,^^28^^,^^47^^,^^48^.

**Scheme 1.**
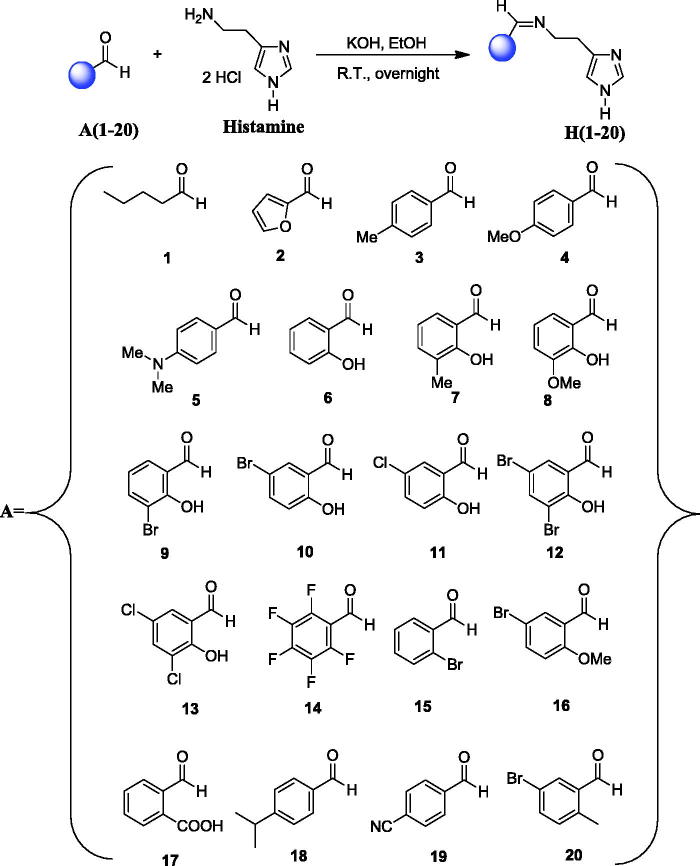
General synthetic route for the synthesis of histamine Schiff bases **H(1–20)**.

A large number of structurally diverse histamine Schiff base derivatives were synthesised according to general synthetic route illustrated in Scheme 1. In order to generate chemical diversity, different substituted aldehydes were chosen, possessing aliphatic, aromatic and heterocyclic moieties, and they were reacted with histamine leading to the new histamine Schiff base derivatives **H(1–20)** (Scheme 1). All the newly synthesised Schiff bases **H(1–20)** were fully characterised by using several analytical and spectral data (see experimental part for details).

### CA activation studies

Schiff base derivatives are known for their important biological applications, especially as efficient and selective CAIs targeting several isozymes among which CA I, IV, and IX isozymes^38–41^^,^^49^, but they were not investigated so far as CAAs. This is the reason why the histamine Schiff base derivatives **H(1–20)** were obtained and assessed as activators of selected CA isoforms, involved in crucial physiologic and pathologic processes. We expect that the nature of aliphatic/aromatic/heterocyclic moiety of the aldehyde reagent will affect the biologic activity of these compounds, and for this reason we used variously substituted aldehydes, in order to explore a wider chemical space within the reported series.

Activation data of five physiologically relevant, cytosolic hCA I, II, and VII, as well as the membrane-anchored (hCA IV) and the transmembrane hCA IX, with compounds, **H(1–20)** and histamine **(HST)** as standard activator, are shown in Table 1. To the best of our knowledge, this is the first study which evaluates the activation profile of histamine Schiff bases on isoforms hCA I, II, IV, VII, and IX. The following SAR can be observed regarding the activation data of Table 1:

**Table 1.** *In vitro* hCA I, hCA II, hCA IV, hCA VII, and hCA IX activation data with histamine Schiff bases **H(1–20)** by a stopped-flow CO_2_ hydrase assay.

	*K*_A_ (µM)^a^				
Comp.**H**	hCA I	hCA II	hCA IV	hCA VII	hCA IX
**1**	18.4	54.2	47.1	20.6	31.4
**2**	1.60	21.7	0.0018	21.9	7.81
**3**	0.85	36.8	1.24	0.81	15.8
**4**	0.24	37.9	1.03	0.052	39.9
**5**	1.17	34.2	2.15	0.061	26.4
**6**	1.02	25.8	1.70	0.012	41.7
**7**	0.94	14.5	1.86	0.038	20.5
**8**	0.80	8.62	0.93	0.006	31.8
**9**	2.83	9.27	0.96	0.012	30.0
**10**	4.14	10.4	1.51	0.007	62.6
**11**	10.7	21.9	36.8	12.6	65.4
**12**	8.52	11.2	18.9	24.6	47.9
**13**	11.2	24.3	25.7	7.13	48.1
**14**	12.7	21.2	33.5	11.4	51.2
**15**	20.4	45.2	29.1	8.23	49.0
**16**	24.3	30.7	20.0	7.16	47.9
**17**	14.1	58.9	24.1	6.26	31.6
**18**	9.64	63.4	28.3	9.51	47.9
**19**	17.8	44.5	29.0	8.44	58.2
**20**	10.4	41.8	30.5	0.78	57.6
**HST**	2.10	125	4.03	37.6	35.2

Histamine **(HST)** has been used as standard activator^42^.

a Mean from three different determinations (errors in the range of 5–10% of the reported values, data not shown).

- The widely abundant cytosolic slow red cell isozyme hCA I was moderately activated by most of the histamine Schiff bases **H(1–20),** with activation constants in the range of 0.24–24.3 µM. Potent hCA I activation has been obtained with the following compounds: **H3**, **H4**, **H7**, and **H8,** which showed *K*_A_s of 0.85, 0.24, 0.94, and 0.80 µM, respectively, which incorporate aromatic moieties with methyl- and/or methoxy moieties at the phenyl ring. The presence of OH, carboxy, or halogeno moieties on the phenyl, as well as the aliphatic or heterocyclic Schiff bases were less effective as hCA I activators (Table 1).
- All compounds reported here **H(1–20)**, were more efficient as hCA II activators compared to histamine **(HST)**, which is a weak activator of this isoform with a *K*_A_ of 125 µM. However, in general, all compounds showed a moderate activation against this isozyme with *K*_A_s ranging between 8.62 µM and 63.4 µM. The best hCA II activators were **H8–10**, and **H12** with *K*_A_s in the range of 8.62–11.2 µM. These derivatives incorporate phenyl moieties substituted with OH, methoxy, and bromine, being thus very diverse of the best hCA II activators discussed above. The compounds **H1**, **H15**, and **H17–20** (incorporating aliphatic, 2-bromophenyl-, as well as 2-COOH, 4-i-Pr, 4-CN, and 2-Me-5-Br-phenyl moieties) were the least effective activators in the series, with *K*_A_s in the range of 41.8–63.4 µM. This structure–activity data show that small differences in the structure of the activator lead to impressive differences of the activating properties, due to the fact that the presence of just one small substituent may lead to clashes or favourable interactions with amino acid residues in the activator binding site, which is situated at the entrance of the cavity, as demonstrated by extensive X-ray structural data for CA-activator complexes^22^^,^^23^^,^^30–36^^,^^50^.
- The membrane-bound isoform hCA IV was activated in a different manner by these compounds. The best activator of this isoform was **H2** with an activation constant of *K*_A_ 1.8 nM. This is the only derivative possessing a furan moiety in the aldehyde fragment of the Schiff base, which confers this excellent, nanomolar affinity for this isoform. It should be mentioned that no X-ray crystal structures of hCA IV in complex with activators are available so far, and the modelling studies of CA-activator complexes were not successful so far, due to the fact that activators do not interact with the metal ion, which leads to important distortion due to the parameterisation of zinc, which dominates over the remaining parts of the complex^51^. Compounds **H3–10** showed better activity (*K*_A_s in the range of 0.93–2.15 µM) than histamine (HST) (*K*_A_ 4.03 µM). The remaining derivatives were medium potency (*K*_A_s in the range of 18.9–47.1 µM, such as **H1**, and **H11–20**).
- The third cytosolic isoform investigated here, hCA VII, was efficiently activated by all compounds reported here with K_A_s in the range of 6 nM to 24.6 µM which all showed better activation potency then starting reference compound histamine **(HST)** (*K*_A_ 37.6 µM). Specifically, derivatives **H4–10** showed nM potency with *K*_A_s ranging from 6 nM to 61 nM against this isoform, which is a key isozyme involved in brain metabolism. They incorporate variously substituted phenyl moieties at the aldehyde fragment of the Schiff base, among which 4-MeO–, 4-Me_2_N–, 2-OH, 2-OH-3-Me-phenyl, etc. (Table 1 and Scheme 1).
- The transmembrane isoform CA IX showed a different activation profile (low micromolar range) compared to the other isoforms discussed here (Table 1). The best hCA IX activator was compound **H2** with *K*_A_ 7.81 µM which also showed the best activation profile against another membrane-bound isoform hCA IV with *K*_A_ 1.8 nM. However **H2** is a medium potency hCA IX activator and highly efficient hCA IV activator. The remaining derivatives showed a micromolar activity with *K*_A_s in the range of 15.8–65.4 µM.
- It is important to mention that the compounds **H4–10** were three orders of magnitude more selective for hCA VII as compared with other isozymes hCA I, II, IV, and IX investigated here. Since hCA VII is a key isoform involved in brain metabolism, cognition^52^, and neuropathic pain^53^, some of these newly synthesised compounds might be investigated as a leads for such neurologic conditions in the search of efficient pharmacologic agents^54^.

## Conclusions

We report here a series of 20 histamine Schiff bases which were synthesised by the reaction of histamine with a large number of substituted aldehydes incorporating aromatic, heterocyclic, or aliphatic moieties. The obtained histamine Schiff bases were investigated as activators of five physiologically relevant CA isozymes, the cytosolic CA I, CA II, and CA VII, as well as the membrane-anchored CA IV and transmembrane CA IX isoforms. All compounds showed a better potency than histamine against isozymes CA I and CA VII with a distinct activation profile and an interesting structure–activity relationship, dependent on the nature of the aldehyde fragment present in the molecule. Many of the compounds showed nanomolar efficacy against isozyme CA VII (*K*_A_s in the range of 6 nM to 24.6 µM) which is a key CA isoform involved in the brain metabolism, cognition, and neuroptic pain. As CAAs may be used in the memory therapy and cognitive neurodegenerative disorders^52^, these histamine Schiff bases reported here may be considered of interest for *in vivo* investigations for possible therapeutic applications of both activators and inhibitors of these enzymes.^17^^,^^52–63^
